# A randomised control trial on oral dydrogesterone *versus* micronized vaginal progesterone pessary for luteal phase support in *in vitro* fertilization cycles

**DOI:** 10.25122/jml-2022-0131

**Published:** 2023-01

**Authors:** Joseph Ifeanyichukwu Ikechebelu, Cyril Chukwudi Dim, George Uchenna Eleje, Ngozi Joe-Ikechebelu, Boniface Chukwuneme Okpala, Princeston Chukwuemeka Okam

**Affiliations:** 1 Department of Obstetrics and Gynaecology, Faculty of Medicine, College of Health Sciences, Nnamdi Azikiwe University, Awka, Nigeria; 2 Life Fertility Centre, Life International Hospital, Awka, Nigeria; 3 Institute of Maternal and Child Health, College of Medicine, University of Nigeria, Ituku-Ozalla, Enugu, Nigeria; 4 Department of Obstetrics and Gynaecology, College of Medicine, University of Nigeria, Ituku-Ozalla, Enugu, Nigeria; 5 Department of Community Medicine & Primary Health Care, College of Medicine, Chukwuemeka Odumegwu Ojukwu University, Awka, Nigeria; 6 Department of Pharmacology & Therapeutics, Faculty of Basic Clinical Sciences, College of Health Sciences, Nnamdi Azikiwe University, Awka, Nigeria

**Keywords:** dydrogesterone, *in vitro* fertilization, luteal-phase support, micronized vaginal progesterone, pessary

## Abstract

This study aimed to evaluate the pregnancy rates, adverse reactions, and medication costs of two luteal phase support regimens: oral dydrogesterone and micronized vaginal progesterone (MVP) pessary in *in vitro* fertilization cycles. A randomized open-label trial with participants randomly assigned to either 400 mg MVP twice daily or 10 mg dydrogesterone three times daily. The primary endpoints were pregnancy rates, and the secondary endpoints included tolerance, miscarriage rates, and medication cost. Per-protocol principle analysis was performed. The baseline characteristics of the 162 participants were similar. Dydrogesterone had statistically similar (p>0.05) positive pregnancy test rates fifteen days post embryo transfer (35.8% *vs*. 32.7%), clinical pregnancy rates at the gestational age of 6 weeks (32.1% *vs*. 28.8%), ongoing pregnancy rates (26.4% *vs*. 23.1%) and miscarriage rates at 14 weeks of gestation (9.2% *vs*. 9.4%) and safety profile to MVP. Dydrogesterone was better tolerated as vaginal itching was significantly more prevalent in the MVP arm (p=0.008). Dydrogesterone is significantly less expensive than MVP pessary. Oral dydrogesterone and MVP pessary had similar pregnancy rates and adverse effects. Dydrogesterone appears more user-friendly and less expensive in cases of luteal-phase support in *in vitro* fertilization cycles.

## INTRODUCTION

Luteal phase support (LPS) is a medical treatment that involves the intake of progestin, progesterone, human chorionic gonadotropin (HCG), or gonadotrophin-releasing hormone (GnRH) agonists to improve the attainment rate of implantation and pre-embryo life thus supporting and complementing the roles of corpus luteum [1]. This practice has become necessary because the luteal function is often compromised during *in vitro* fertilization and embryo transfer (IVF-ET) cycles. Previous reports have shown a considerable drop in conception rates among women without LPS undertaking IVF-ET [2, 3].

A recent Cochrane meta-analysis has reported different methods, prescriptions, and ways of administering LPS in assisted reproductive technologies (ART) [1]. Compared to oral administration, parenteral or vaginal administration of progesterone appears not to predispose the progesterone complex to the substantial energy conversion to its 5α and 5β products often seen in oral administration [4]. Another possible downside of oral administration of progesterone is that it is subject to pre-systemic pre-liver and intra-liver effects.

To overcome these potential limitations, Duphaston, a synthetic form of the hormone progesterone known as dydrogesterone, was produced. Duphaston is chemically identical to natural progesterone, but the methyl group in carbon 10 is situated in the alpha position rather than the beta position in natural progesterone [1, 4]. As a result of the different compositions seen in DYD, it makes it more effective and stable when administered orally. This increases patients' amenableness and associated low local adverse effects without affecting pregnancy rates [5]. Generally, administering the drug through the mouth is easier and could make it most acceptable [1]. However, compared to the oral route, the vaginal route often results in higher uterine concentrations and could be very uncomfortable or washed off in women with vaginal bleeding [1].

Previous studies have documented the safety, efficacy, and tolerability of DYD [1, 6–9]. For instance, it has been reported that DYD has no male characteristic predisposition property on the fetus and does not hinder the placental development of progesterone. Several studies specifically reported no congenital anomalies associated with its use in IVF practice [8–10].

Nevertheless, there are various reports on pregnancy outcomes following the administration of DYD *versus* vaginal progesterone in ART cycles. Consequently, this study aimed to fill the gap in our understanding regarding these differences. While Enatsu *et al*. [11] reported superior body adsorption with fewer comparative changeability when compared to oral progesterone, Barbosa *et al*. [12] and Tournaye *et al*. [9] showed that DYD is not inferior to micronized vaginal progesterone (MVP) with the later as the standard of care. Women's satisfaction with MVP treatment has been affected by the irritating vaginal discharge and itching associated with its use. Additionally, newer reports have revealed that MVP alters the local microbiome and associated endometrial changes in the microorganism environment of the uterus. These changes could lead to progesterone-unaffected uterine syndrome [13, 14]. Women have no choice but to continue its use since it is required for a successful outcome with the IVF cycle.

Therefore, it is expected that oral medications with similar outcomes to vaginal medications would be more acceptable to women. The use of oral DYD is as effective as our current standard of care – MVP pessary. Given the pervasive use of MVP and the greater number of *in vitro* fertilization cycles achieved globally, a new systematic review and meta-analysis surmised that further studies evaluating participant acceptability, costs, safety, and efficacy are justified [15]. This study aimed to determine and compare pregnancy rates, adverse reactions, and medication costs of two luteal phase support (LPS) regimens: oral DYD and MVP pessary in IVF-ET cycles.

## MATERIAL AND METHODS

### Study location

The study was conducted in Anambra State in Southeast Nigeria, predominantly inhabited by the Igbo. The two IVF centers used in this study are in Awka and Nnewi, Anambra State of Nigeria. The only other IVF center in the Southeast zone is located at Enugu. Infertility rates are high in the area [16], with male infertility as the leading cause in southeast Nigeria [17–19]. However, this challenge is perceived differently in each couple experiencing infertility [16].

### Study population

Life International Hospital Awka and Life Specialist Hospital Nnewi, Nigeria, are referral centers for IVF and thus receive clients from all over southeast Nigeria and some Igbos in the diaspora. The study population comprises infertile couples referred for IVF treatment from the study centers or other health facilities in southeast Nigeria.

### Study centers

This study was conducted at the fertility centers of Life International Hospital, Awka (LIHA), and Life Specialist Hospital Nnewi (LSHN), both in Anambra State, Nigeria. Both are multi-specialist hospitals (ObGyn, Surgery, Internal Medicine, and Paediatrics) with consultants in each of the specialties and 50-bed capacity (LIHA) and 30-bed capacity (LSHN), respectively. IVF services started at LSHN in August 2010 and LIHA in February 2018. Both centers offer assisted reproductive technology services and receive referrals from the South-East region and the rest of Nigeria, including Igbos in the diaspora.

### Study design

A randomised open-label study comparing oral DYD (Duphaston^®^; Abbot B.V., The Netherlands) with the standard-of-care in Nigeria – micronized vaginal progesterone pessary (Cyclogest^®^; Actavis, UK) in LPS for women undergoing IVF-ET treatment with stimulated cycles or donated oocyte.

### Inclusion criteria

Infertile couples scheduled for IVF-ET cycles with self or donated ovum. Women with stimulated cycles [self-ovum] and aged between 20 and 40 years with FSH level <10 IU/l were randomized. Women using donated ovum, less than 55 years of age, peri-menopausal, or menopausal were randomized.

### Exclusion criteria

Women scheduled for self-ovum cycles with severe adenomyosis, symptomatic uterine fibroids, advanced endometriosis, and chronic hepato-renal disease were excluded. Women using donated ovum with multiple medical co-morbidities that may be possibly aggravated by pregnancy were excluded.

### Participant recruitment

*In vitro* fertilization cycles were done in lots of 20–30 couples per cycle to enhance existing assets and workforce. After counseling and written consent were completed, participants were referred for *in vitro* fertilization and enlisted by the IVF unit nurses/counselors before starting downregulation. Liver function tests were done pre- and post-therapy to assess possible systemic adverse drug reactions. The IVF-ET procedure was carried out as previously documented by Ikechebelu *et al*. [20]. The details are described below.

### Down-regulation and ovarian stimulation

The levels of follicle-stimulating hormone (FSH), luteinizing hormone (LH), and oestradiol on menstrual days 2–5 were measured by the in-house embryologist pre-intervention to assess the ovarian "backups" using automated "mini Vida" hormone assay machine. A transvaginal ultrasound was done before starting the GnRH agonist on the 21^st^ day of the monthly cycle.

Long-protocol down-regulation procedure started on day 21 with an injection of 3.6 mg goserelin (Zoladex^®^, AstraZeneca, Cheshire, United Kingdom) or day-to-day doses of 0.5 mg buserelin acetate (Suprefact^®^, Hoechst AG, Frankfurt, Germany), self-administered subcutaneously at home (or at the hospital by the unit nurses). This was stopped when ovarian suppression was achieved. Confirmation was done when the thickness of the endometrium was <4 mm on ultrasound before FSH administration or the oestradiol level was less than 50 pg/ml. Ovary stimulation was started for women with optimal down-regulation at day 14 to day 28 of treatment according to the center schedule. Women who failed to down-regulate were moved to the next scheduled group. No down-regulation was required for menopausal women.

For women on self-ovum treatment and the ovum donors, multiple-follicular growth stimulation was achieved using human-menopausal-gonadotrophin (Menopour^®^, Ferring, Germany R) given in age-adjusted dose. Therefore, the doses were 225 IU daily for women younger than 34, 300 IU daily for those aged between 35 and 38, and 375 IU daily for those older than 38. In order to ensure ovarian monitoring, a transvaginal ultrasound was done, and the endometrium was monitored between the 6^th^ and 9^th^ day of stimulation by the researcher. A pre-human-chorionic-gonadotropin (pre-hCG) scan was done on day 11 to confirm follicular maturation showing at least two follicles reaching 17 mm or more for ovarian diameter. Then ovulation was initiated using a single dose of 10,000 IU (or 5,000 IU if suspected of ovarian hyper-stimulation) of hCG (Diclair-HP hCG^®^, R Germany). Oocyte retrieval was then scheduled 34 hours after the hCG injection.

Women requiring ovum donation were started on oestradiol valerate 4 mg three times daily (Oestrafert^®^ West-Coast Pharmaceutical Works Ltd, Gujarat, India) to grow the endometrium.

### Oocyte retrieval

Both study centers grouped the randomized participants and had the oocyte recovery, insemination/embryo transferal. The researcher (who holds a certificate training in ART from Ottawa, Canada, and is an established fertility expert with 9 years of experience in IVF practice) performed the retrieval and transfer with the aid of transvaginal ultrasound to guide the aspiration 34–36 hours after hCG administration using a 17-gauge aspiration needle (Wallace^®^, Smith Medical International Ltd., Mexico, USA) and regulated suction pro equipment.

### In vitro fertilization and embryo transfer (ET)

Only fresh embryo transfers were used in the study. Oocytes were inseminated with prepared sperm specimens [20, 21]. These were performed by 2 embryologists. On Day 3 or 5, ET was performed by the researcher when the embryos were at the 6–8 cell or blastocyst stage.

### Luteal phase support

The LPS was started on the exact date of the oocyte retrieval and sustained until 14 weeks of gestation in fruitful cycles.

### Randomization and allocation sequence

162 consenting participants were randomized [1:1 ratio, blocks of 4] into group A, oral dydrogesterone group, and group B, or vaginal cyclogest group. Two equal sets of computer-generated numbers (81 per set) representing each group of the study were generated by an independent statistician. Brown opaque envelopes were numbered consecutively from 1 to 162. Each numbered envelope contained the study group corresponding to its number and was sealed. The envelopes were arranged serially and secured in a locked cupboard at the hospital in Awka and managed by a nurse blinded to the study. Once a woman gave consent for the study and was assigned a serial number, the nurse was reached to open the envelope corresponding to the serial number and announce the group concealed inside for participants allocation.

### Blinding of participants, personnel, and outcome assessors

Blinding of participants, personnel, and outcome assessors was not possible due to the open-label nature of the study.

### Outcome measures

The primary endpoints included the clinical pregnancy rate. The secondary endpoints included positive pregnancy test rate, ongoing pregnancy rate, tolerance rate, miscarriage rate, medication cost, and presence of adverse effects such as derangement in aspartate transaminase (AST), alanine transaminase (ALT) and alkaline phosphatase (ALP) parameters.

In this study, we defined clinical pregnancy at the ultrasound scan completed at 6 weeks showing the presence of a viable fetus. Miscarriage is the loss of a fetus before the 20^th^ week of pregnancy. The presence of at least one viable fetus at 14 weeks gestation was classified as ongoing pregnancy. For this study, women were followed up until 14 weeks gestation.

Biochemical pregnancy was defined as serum β-hCG level presence on day 15 post-ET, while clinical pregnancy was confirmed by ultrasound detection of a viable fetus at 6 weeks post-ET. The aspartate transaminase (AST), alanine transaminase (ALT), and alkaline phosphatase (ALP) parameters were assessed pre-and post-therapy for monitoring adverse effects.

### Sample size estimation

Sample size estimation was derived from a formula by Fleiss [22] and based on the previous study by Ikechebelu *et al*. [20], which reported a 27% clinical pregnancy rate for participants who used micronized vaginal progesterone and a 54% clinical pregnancy rate for those who used oral dydrogesterone (*i.e*., twice that of micronized vaginal progesterone). Thus, considering 90% power, 10% attrition rate, and 5% error, the sample size was 81 cases in each group.

### Data analyses

Data entry and analyses were carried out using Statistical Package for Social Sciences (SPSS) version 23, IBM Company, USA. The analysis was done per protocol. Categorical data were treated as numbers and percentages, and continuous data as mean and standard deviations (mean±SD). For inferential statistics, the Chi-square (X^2^) or Fisher's exact test was used to compare categorical variables, and the independent student's t-test was used to compare continuous variables in two groups. For this study, a P-value of less than 0.05 was considered statistically significant.

## RESULTS

### Description of study flow

**Figure 1:**
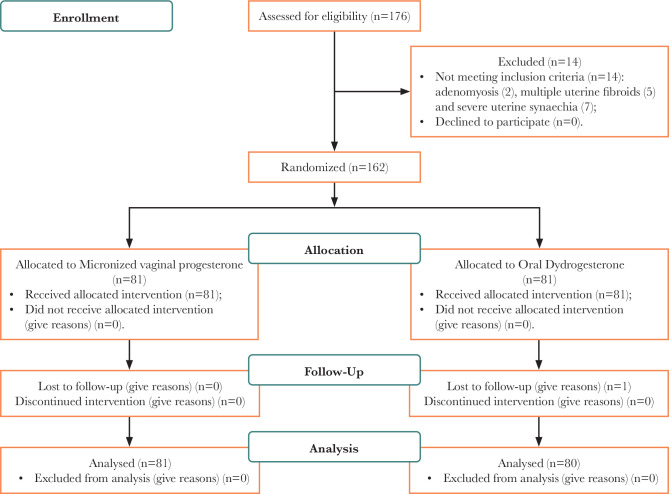
Flow chart of the study participants who underwent fresh embryo transfer.

176 women were screened for this study between March 2019 and September 2019. Fourteen women did not meet the inclusion criteria: adenomyosis (n=2), multiple uterine fibroids (n=5), and severe uterine synechiae (n=7). Therefore, 162 women were randomized and participated in the trial, of which 81 women were allocated to the oral dydrogesterone (DYD) group and 81 women to the micronized vaginal progesterone (MVP) group (Figure 1).

### Participants' basic characteristics and cause of infertility

As shown in Table 1, the mean age of participants in the DYD group (37.7±6.5, range 32–47 years) was similar to that of the MVP group (37.8±6.1, range 30–49 years) (p=0.935). There were no significant differences in other baseline demographic and clinical data between the two study groups, including educational status, infertility duration, mean endometrial thickness, day of embryo transfer, number of embryos transferred (Table 1), and the cause of infertility (Table 2).

**Table 1:** Baseline demographic and clinical characteristics of the participants.

Parameter	Oral DYD (n=81) Frequency (%)	MVP Pessary (n=81) Frequency (%)	P-value
**Age ranges (years)**			
25–29	5	5	1.000
30–34	7	6	
35–39	23	24	
40–44	21	20	
45–49	23	23	
50–54	2	3	
**BMI (mean±SD)**	27.7±3.2	27.3±5.4	0.635
**Educational level**			
Primary	-	-	-
Secondary	38 (46.9)	40 (49.4)	0.846
Tertiary	43 (53.1)	41 (50.6)	
**Duration of infertility (years)**			
≤5 years	35 (41.5)	31 (38.3)	0.690
>5 years	46 (58.5)	50 (61.7)	
**Endometrial thickness (mean±SD) (n=81)**	6.4±0.8	6.3±0.7	0.498
**No of oocyte retrieved (mean±SD) (n=81)**	7.7±1.4	7.8±1.7	0.743
**No of embryo transferred (mean±SD) (n=81)**	2.6±0.8	2.5±0.9	0.527
**Day of embryo transfer (mean±SD) (n=81)**	3.0±1.6	3.0±1.5	1.000

Values are expressed as number (percentage) of women.

**Table 2:** Causes of infertility.

Parameter	Oral DYD (n=81) Frequency (%)	MVP Pessary (n=81) Frequency (%)	P-value
**Cause of infertility**			
Tubal factor	29 (35.8)	28 (34.6)	0.680
Sperm factor	25 (30.9)	25 (30.9)	0.509
Both sperm & tubal factor	7 (8.6)	6 (7.4)	1.000
Menopausal factor	3 (3.7)	3 (3.7)	0.716
PCOS	2 (2.5)	5 (6.2)	1.000
Uterine synaechia	3 (3.7)	5 (6.2)	0.678
Endometriosis	2 (2.5)	2 (2.5)	0.678
Unexplained	7 (8.6)	5 (6.2)	1.000
Others	3 (3.7)	2 (2.5)	1.000

Others – seeking male child (n=1), adhesion factor (n=1), age related factor (n=2); expressed as number (percentage) of women.

### Pregnancy rates in the study groups

The details of the primary and secondary outcome measures relating to the pregnancy rates are shown in Table 3.

**Table 3:** Distribution of pregnancy rates and miscarriage rates in the study groups.

Pregnancy rates	Oral DYD (n=81) Frequency (%)	MVP Pessary (n=81) Frequency (%)	RR (95% CI)	P-value
**Positive pregnancy test**	19 (35.8)	17 (32.7)	1.07 (0.72–1.58)	0.837
**Clinical pregnancy**	17 (32.1)	15 (28.8)	1.08 (0.72–1.61)	0.833
**Ongoing pregnancy**	14 (26.4)	12 (23.1)	1.09 (0.72–1.66)	0.822
**Miscarriage**	7 (8.6)	8 (9.9)	0.99 (0.52–1.90)	>0.999

Values are expressed as number (percentage) of patients.

### Incidence of miscarriage within study groups

The incidence of miscarriage in the DYD group was 9.4% (5/52), while in the control (MVP) group, it was 9.2% (5/53). The observed difference was not statistically significant (RR=1.0, 95% CI: 0.31–3.31, p=1.0) (Table 3).

### Side effects (tolerability) of drugs within study groups

The frequency of vaginal itching was significantly higher in the MVP group. Other side effects observed and the adverse reactions are displayed in Table 4.

**Table 4:** Tolerance rate and liver function test parameters.

Parameter	MVP Pessary (n=81) Frequency (%)	Oral DYD (n=81) Frequency (%)	RR (95% CI)	P-value
**Tolerance rate (n=81)**				
V. itching	20 (24.7)	4 (4.9)	1.90 (1.39–2.60)	*0.008
Nausea (N)	5 (6.2)	11 (13.6)	0.57 (0.22–1.50)	0.201
Vomiting (V)	13 (15.1)	13 (16.0)	0.99 (0.58–1.68)	1.000
N & V	8 (9.9)	9 (11.1)	0.63 (0.30–1.31)	0.174
V. itching & V	28 (34.6)	5 (18.5)	1.43 (0.99–2.07)	0.113
None	13 (16.0)	26 (32.1)	0.60 (0.33–1.09)	0.076
**Liver Function test (n=81)**				
AST (U/L)				
Baseline	12.1±5.1	11.5±5.1	ŧ-0.904	0.368
After treatment	9.9±3.0	9.7±4.2	ŧ-0.280	0.780
ALT (U/L)				
Baseline	10.1±4.8	10.3±4.8	ŧ 1.533	0.128
After treatment	8.9±3.8	9.1±5.6	ŧ 0.428	0.670
ALP (U/L)				
Baseline	40.8±14.3	38.9±14.9	ŧ -0.597	0.552
After treatment	38.7±10.1	37.3±13.2	ŧ -0.610	0.543

AST – Aspartate transaminase; ALT – Alanine transaminase; ALP – Alkaline phosphatase; V. itching – Vaginal itching; ŧ – t-test or Z-test; RR – Risk ratio; 95%CI – 95% confidence interval. Values are expressed as number (percentage) of patients.

### Cost of drugs within study groups

The study drugs were procured from official drug distributors in Nigeria at their prevailing price. This cost was borne by the researcher and did not affect the cost of IVF treatment for the participants in this study. The analysis of the cost of treatment is presented in Table 5 and shows that DYD is significantly less expensive. The user-friendliness was assessed based on the routes of administration since one agent was administered orally while the other was administered vaginally with its associated vaginal examination. Participants had a higher rating for user-friendliness when using oral dydrogesterone (DYD) compared with micronized vaginal progesterone (MVP) pessary.

**Table 5:** Cost analysis of drugs used in the study.

Drug	Cost per pkt (N)	No of Tab per pkt	Cost per tab N (USD)	No. of Tab till 14d pET	Cost of Tx till 14d pET N (USD)	No. of Tab till 14w GA	Cost of Tx till 14w GA N (USD)
**Duphaston**	2,300	20	115 ($0.3)	57	6,555 ($18)	309	35,535 ($99)
**Cyclogest**	8,500	15	567 ($1.6)	38	21,546 ($60)	206	116,802 ($325)

* all figures in Naira (Nigeria): Official exchange rate $1 (US)=N360. Price as at January 2019. pkt – packet; Tx – Treatment; w – week; pET – Post Embryo Transfer; N – Nigerian Naira; USD – United States Dollars.

## DISCUSSION

In this randomized study, oral dydrogesterone (DYD) was comparable with micronized vaginal progesterone (MVP) pessary regarding chemical pregnancy rate, clinical pregnancy rate, ongoing pregnancy rate, and miscarriage rates. Regarding the tolerability profiles, the two agents are comparable, except that the frequency of vaginal itching was significantly higher in the MVP group (p=0.008). Nevertheless, there was no difference in adverse effects between the study groups.

In previous studies on LPS, such as Lotus I [9] and Lotus II [7], DYD had comparable efficacy with MVP. However, while the Lotus I trial revealed that DYD was not inferior to MVP pessary in conception frequencies at 12 weeks of gestation after LPS, the Lotus II trial revealed that DYD was not second-rate to MVP liniment in fresh-cycle IVF. Our study has further demonstrated the findings of these previous studies in a Nigerian population.

We also found the clinical conception frequencies in the DYD group comparable but lower to that of a recent Lotus II study [7], which documented a clinical conception frequency of 38.7%, whereas we obtained a clinical pregnancy rate of 32.1%. Similarly, regarding the MVP group, our clinical conception frequency was also lower (28.8%) compared with 35.0% in Griesinger *et al*. [7] study. The difference in these findings is probably a result of different inclusion criteria and our study population consisting only of infertile women with conventional IVF. The study of Griesinger *et al*. excluded women with four or more IVF shots or a prior history of recurrent miscarriage [7].

A recent study of the categorized population in China observed a variation of 9.4% favoring DYD. However, current conception frequencies at the gestation age of 12 weeks of 61.4% and 51.9% for DYD and MVP gel arms, respectively, were observed [23]. Our finding on the ongoing conception rate was lower than the two groups (26.4% for the DYD group *vs*. 23.1% for the MVP group). Yang *et al*. study used 12 weeks, while we used 14 weeks of gestation in defining ongoing pregnancy [23], which may have contributed to the observed difference in both studies. Another possible factor in the overall outcomes between our index study and that of the Lotus II study was the difference in the mean ages and body mass index between the two studies. In the Lotus II studies, the mean ages were 31.8±4.4 *vs*. 31.6±4.6 years for DYD and MVP gel groups, respectively, while in ours, the average age was higher at 37.8±6.1 and 37.7±6.5 years respectively. Similarly, in Lotus II studies, the mean body mass index was 23.1±3.1 *vs*. 23.1±3.0 kg/m^2^ for DYD and MVP gel groups, respectively, while in ours, the average body mass index was 27.7±3.3 and 27.3±5.1 kg/m^2^, respectively [23].

Although pregnancy loss incidences were not studied as straight endpoints in the Lotus I and Lotus II trials and the recent Griesinger *et al*. [7] study, our research demonstrated that the two treatment groups have two comparable miscarriage rates of 9.2% and 9.4% for DYD and MVP groups respectively. Similar to our findings on the role of LPS among participants undergoing IVF, Chakravarty *et al*. [24] did not reveal any significant dissimilarities in conception incidences and pregnancy loss incidences between women receiving DYD and MVP. Ganesh *et al*. [25] came to similar conclusions. Ganesh *et al*. evaluated DYD, progesterone gel, and MVP for LPS and reported no differences among the three groups in overall conception and pregnancy loss incidences [25].

Despite the similarities in efficacy, we also found no difference concerning the adverse effects, except for the tolerance rate, which was significantly lower in the MVP group on account of vaginal itching. This finding is understandable since the MVP pessary is administered vaginally as described in the study protocol. Despite the non-significant difference in efficacy, we did not see any significant occurrence of adverse effects rate in the two groups. The aspartate transaminase (AST), alanine transaminase (ALT), and alkaline phosphatase (ALP) parameters did not significantly change pre- and post-therapy. After pregnancy support with DYD or MVP, the ongoing conception incidence was higher than the live birth rate documented in a previous study at one of the hospitals [19].

In this study, the protocol requires that the luteal phase support drugs be administered for 14 weeks of gestation. However, the exact prime dose and the timing of the stoppage of the luteal phase support with progestogens during pregnancy have remained unanswered [26].

Although we utilized only fresh embryo transfer during the study, a recent study by Le *et al*. [27] revealed that the freeze-all approach was linked to better cumulative live birth rates in regular and over-responders, although not observed for non-optimal or failed responders. Another study by Cirillo *et al*. [28] revealed that fresh-embryo-transfers produced marked neonatal and maternal complications compared to frozen-embryo-transfer. A recent Cochrane review evaluated the adverse effects of the freeze-all approach compared to the straight IVF/ICSI approach and concluded that there was moderate-certainty information revealing that one approach was inferior to the others [29].

The cost of the LPS drugs used in this study reveals that DYD is about three times less expensive than the MVP pessary. This difference is significant and may be a key factor in women's choice, as anything that will reduce the cost of IVF, especially in low resource environment, is welcomed. This is important, considering that couples in most low-income settings pay for IVF treatment on an out-of-pocket basis as the treatment is not covered by all available health insurance policies [26]. There is an ongoing strategy to introduce low-cost IVF with natural cycles and eliminate the high cost of ovarian stimulation. This effort, combined with the use of DYD for LPS, may be the ultimate strategy for low-cost IVF in low-resource settings.

This study appears to be the first study comparing DYD and MVP pessary for women undergoing IVF-ET in terms of cost analysis. The focus of the analysis regarding the follow-up of participants was the ongoing conception rate, but closer clinical attention could be paid to the live birth rate. The protocol was restricted to pregnancy, tolerance, and miscarriage rates and did not consider live birth rates. We could not assess the live birth rates not only due to the duration of the study but also because some women did not deliver in the hospital as they came from referrals within and outside the country. Live birth rate and congenital disabilities were, therefore, not included in our outcome. Our study was an "open-label" study design as we could not provide any placebo medication for either of the intervention agents. This might have augmented the risk of bias in the outcomes described in the current study. Additional exploration is needed in our environment to investigate the value of the two agents concerning live birth rates. Additionally, we made our cost analysis relatively simple, at least to improve the understanding of our global readership [30]. Although a sample size calculation was determined at 81 cases in each group, we acknowledge that our sample size was very small to detect clinically relevant differences because the study was not sufficiently powered to detect a 15% difference in clinical pregnancy.

## CONCLUSION

The incidence of positive pregnancy tests, clinical pregnancies, and ongoing pregnancy rates in women that used oral DYD for LPS during IVF–ET was similar to that of women who used MVP pessary. Similarly, the tolerability of the drugs did not differ between the two groups except for vaginal itching, which was expressively greater in the MVP when compared to the DYD group. It appears less expensive to use DYD than MVP for LPS. When available, oral DYD can safely be used for LPS in the IVF-ET cycles. This study validates oral DYD as a feasible alternative to MVP pessary because of its similar pregnancy rates and fewer adverse effects. Since the oral route is user-friendly and DYD appears cheaper, DYD may continue to replace MVP pessary as the LPS of choice among women undergoing IVF-ET.

## Data Availability

The datasets used and/or analyzed during the current study are available from the authors upon reasonable request.

## Personal thanks

The authors appreciate the help of the staff of Life International Hospital Awka and Life Specialist Hospital Nnewi and the participants involved in the trial. The study was developed from the lead author's MSc Thesis, "Comparison of oral dydrogesterone and micronized vaginal progesterone pessary for luteal phase support in *in vitro* Fertilization cycles at Fertility Centres in Anambra State, Nigeria: A randomised control trial", presented for the MSc Human Reproduction & Women Health at the Institute of Maternal and Child Health, College of Medicine, the University of Nigeria Ituku-Ozalla, Enugu, Nigeria; 7 October 2019.
